# 848. Stratified Morbidity in ≥ 15% TBSA Pediatric Burns: A Cohort Study for Prognostication and Benchmarking

**DOI:** 10.1093/jbcr/irag033.318

**Published:** 2026-04-06

**Authors:** Ryan T Davis, Ryan D Rosen, Darina N Malinova, Rachael M Galvin, Kelli N Patterson, Elika Ridelman, Justin D Klein, Christina M Shanti

**Affiliations:** Wayne State University School of Medicine, Children's Hospital of Michigan Burn Center, Detroit, MI; Detroit Receiving Hospital Burn Center, Ferndale, MI; Wayne State University School of Medicine, Children's Hospital of Michigan Burn Center, Detroit, MI; Wayne State University School of Medicine, Children's Hospital of Michigan Burn Center, Detroit, MI; Wayne State University School of Medicine, Children's Hospital of Michigan Burn Center, Detroit, MI; Wayne State University School of Medicine, Children's Hospital of Michigan Burn Center, Detroit, MI; Wayne State University School of Medicine, Children's Hospital of Michigan Burn Center, Detroit, MI; Wayne State University School of Medicine, Children's Hospital of Michigan Burn Center, Detroit, MI

## Abstract

**Introduction:**

Pediatric burns remain a major cause of morbidity, yet modern complication rates and resource utilization benchmarks are poorly defined. Intra-abdominal sequelae such as abdominal compartment syndrome (ACS), necrosis, and perforation are rarely documented systematically in children. We sought to establish contemporary complication rates, resource utilization patterns, and abdominal outcomes in severe pediatric burns.

**Methods:**

We retrospectively reviewed pediatric patients (≤18 years) with burns ≥15% total body surface area (TBSA) admitted to a regional pediatric burn center between 2015 and 2024. Demographics, burn characteristics, hospital length of stay (LOS), ventilator use, systemic complications, and mortality were abstracted. Patients were stratified by TBSA bands (15–19.9%, 20–29.9%, 30–49.9%, ≥50%). Nonparametric and Fisher’s exact testing compared outcomes.

**Results:**

Ninety-four patients met inclusion criteria (median age 2.0 years, TBSA 27%). Resource use rose stepwise with TBSA (median LOS 11 to 55 days, *p*=.004; ventilator days *p*<.001; ventilation required *p*<.001). Inhalation injury also increased significantly in ≥30% TBSA patients (*p*=.0005). Complications clustered in ≥30% TBSA: pneumonia (*p*=.010), ARDS (*p*<.001), AKI (*p*<.001), and shock (*p*<.001). Mortality was 4.3% (4/94), confined to ≥30% TBSA (*p*<.001). Abdominal complications occurred in 4 patients (4.3%), including abdominal compartment syndrome (n = 4), intestinal necrosis (n = 1), and bowel perforation (n = 1); two patients had multiple events. Pooled intra-abdominal complications were significantly more common in ≥30% TBSA (*p*=.008).

**Conclusions:**

High-TBSA burns are associated with substantial systemic, respiratory, and abdominal morbidity. Abdominal sequelae, although rare, were systematically captured and may be underrecognized in this population.

**Applicability of Research to Practice:**

This study offers complication benchmarks and highlights the need for surveillance for intra-abdominal events in large TBSA burns. These findings may inform fluid resuscitation strategies, guide family counseling, and support prognostication in high-TBSA pediatric burns. Stratified complication data may also aid in developing early warning tools and multicenter collaborative efforts.

**Funding for the study:**

N/A.

